# The Influence of Bacterial Diet on Fat Storage in *C. elegans*

**DOI:** 10.1371/journal.pone.0007545

**Published:** 2009-10-21

**Authors:** Kyleann K. Brooks, Bin Liang, Jennifer L. Watts

**Affiliations:** School of Molecular Biosciences, Washington State University, Pullman, Washington, United States of America; Buck Institute for Age Research, United States of America

## Abstract

**Background:**

The nematode *Caenorhabditis elegans* has emerged as an important model for studies of the regulation of fat storage. *C. elegans* feed on bacteria, and various strains of *E. coli* are commonly used in research settings. However, it is not known whether particular bacterial diets affect fat storage and metabolism.

**Methodology/Principal Findings:**

Fat staining of fixed nematodes, as well as biochemical analysis of lipid classes, revealed considerable differences in fat stores in *C. elegans* growing on four different *E. coli* strains. Fatty acid composition and carbohydrate levels differ in the *E. coli* strains examined in these studies, however these nutrient differences did not appear to have a causative effect on fat storage levels in worms. Analysis of *C. elegans* strains carrying mutations disrupting neuroendocrine and other fat-regulatory pathways demonstrated that the intensity of Nile Red staining of live worms does not correlate well with biochemical methods of fat quantification. Several neuroendocrine pathway mutants and eating defective mutants show higher or lower fat storage levels than wild type, however, these mutants still show differences in fat stores when grown on different bacterial strains. Of all the mutants tested, only *pept-1* mutants, which lack a functional intestinal peptide transporter, fail to show differential fat stores. Furthermore, fatty acid analysis of triacylglycerol stores reveals an inverse correlation between total fat stores and the levels of 15-methylpalmitic acid, derived from leucine catabolism.

**Conclusions:**

These studies demonstrate that nutritional cues perceived in the intestine regulate fat storage levels independently of neuroendocrine cues. The involvement of peptide transport and the accumulation of a fatty acid product derived from an amino acid suggest that specific peptides or amino acids may provide nutritional signals regulating fat metabolism and fat storage levels.

## Introduction

Many components regulating human metabolism are conserved in the nematode *C. elegans*, including biochemical pathways of fat, carbohydrate, and protein synthesis and breakdown as well as neuroendocrine regulators of growth, reproduction, and metabolism [1]–[3]. *C. elegans* mutants that influence fat metabolism often affect aging, for example, mutants in insulin/IGF and TGF-β pathways mediating the response to food signals have longer lifespans (reviewed in [4], [5]). Neuroendocrine ligands are produced in response to food and other inputs in specific sensory cells, resulting in activation of signal transduction pathways that subsequently activate or repress transcription factors in peripheral cell types. These transcription factors regulate gene expression of various genes involved in metabolism, longevity, and developmental fate decisions [6]–[8].

In laboratory settings, *C. elegans* feeds on bacterial lawns growing on agar plates. *E. coli* strains that have been commonly used by *C. elegans* researchers include OP50, a strain chosen by Sydney Brenner because it forms a thin lawn that allows for optimal visualization of *C. elegans* development [9]; DA837, a strep-resistant strain derived from OP50, previously used in studies of food preference and satiety [10], HB101, a B x K12 hybrid that forms a visibly thicker lawn than OP50 or DA837 [11], and HT115(DE3), a K12-derived RNAse III minus strain used for RNAi feeding experiments [12]. Several studies have shown that the nematodes prefer certain bacterial strains and will leave one food source to seek out other sources, suggesting that *C. elega*ns hunt for food that best supports growth [10], [13]. However, it is not known whether particular bacterial diets affect the metabolism of the worms.

We used fat staining of fixed nematodes and biochemical lipid analysis to demonstrate considerable differences in fat stores in *C. elegans* feeding on various *E. coli* strains. Analysis of macronutrients in the *E. coli* strains revealed differences in carbohydrate content and fatty acid composition among the strains, although these differences are not likely to be causative for the differential fat storage in the nematodes. Surprisingly, all of the neuroendocrine mutants tested in this study showed fat storage differences when feeding on two of the *E. coli* strains, indicating that these pathways are not necessary for differential fat storage. However, a mutant carrying a deletion in a gene encoding an intestinal peptide transporter, *pept-1*, stores equally high levels of fat regardless of its dietary bacteria. In addition we identified a significant inverse correlation with a specific fatty acid, 15-methylpalmitic acid (C17iso), derived from leucine catabolism and fat stores in *C. elegans*.

## Results

### Fat storage levels in *C. elegans* depend on the dietary bacterial strain

**Figure 1:**
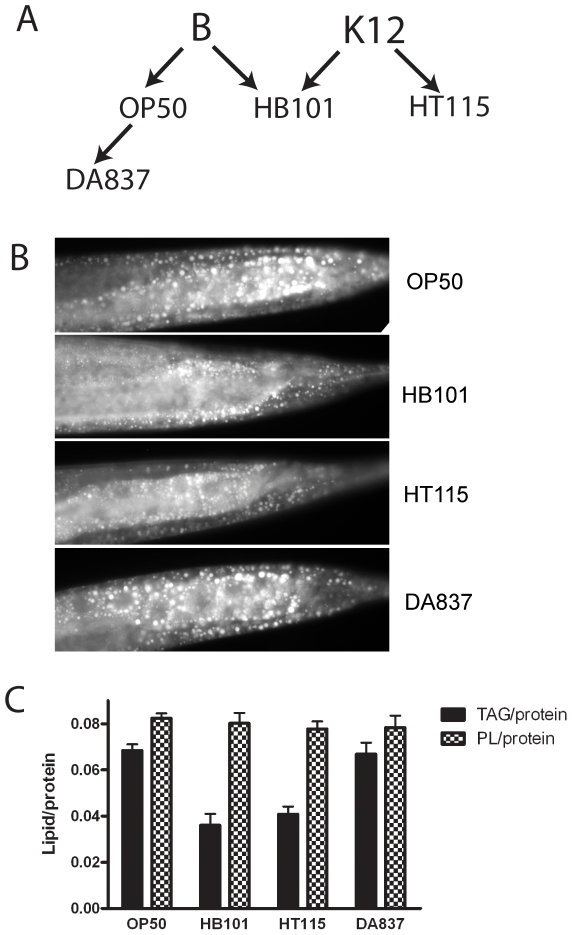
Dietary *E. coli* influences fat storage in *C. elegans*. (A) Ancestral relationship between four *E. coli* strains used in this study. OP50 and DA837 are derived from *E. coli* strain B, HB101 is a B x K12 hybrid, and HT115(DE3) is derived from *E. coli* K-12. (B) Fat stores in *C. elegans* depend on the dietary *E. coli* strain. Young adults were fixed with paraformaldehyde and stained with Nile Red. (C) Lipids were extracted from young adult *C. elegans* grown on four *E. coli* strains. Total protein was determined using an aliquot of the nematode pellet used for lipid extraction. Lipid extracts were separated into phospholipids and TAGs using thin layer chromatography and quantified using gas chromatography. Error bars are SEM, n = 3−5 independent growths. The fatty acids in phospholipid fractions did not vary but the relative amount of fatty acids found in TAG fractions varied up to 2 fold depending on the dietary *E. coli* strain.

To investigate how *E. coli* diets affect fat storage in *C. elegans*, we examined fat stores in worms feeding on four *E. coli* strains that are commonly used by *C. elegans* researchers: OP50 [9]; DA837 [10], HB101[11] and HT115 (DE3) [12] (Figure 1A). Staining live worms with the lipophylic dye Nile Red did not reveal differences in staining pattern or intensity in worms feeding on these particular *E. coli* strains, however, when worms were first fixed in paraformaldehyde [6], and then stained with Nile Red, we found that the size and intensity of stained lipid droplets varies depending on the particular *E. coli* strain upon which the worms are feeding (Figure 1B). *C. elegans* raised on OP50 and DA837 lawns showed larger lipid droplets and more intense staining than those raised on HB101 or HT115. We extracted lipids from young adult nematodes growing on all four bacterial strains, and found that phospholipid levels did not vary in worms feeding on the four *E. coli* strains, but triacylglycerol (TAG) levels varied greatly, with double the amount of fatty acids in TAG fractions in worms grown on OP50 compared to HB101. Young adults raised on DA837 had similarly high levels of TAGs as OP50, while worms raised on HT115 had reduced TAG levels, nearly as low as worms grown on HB101 (Figure 1C).

### Nile Red staining of fixed worms is a better indicator of fat stores than Nile Red staining of live worms

**Figure 2:**
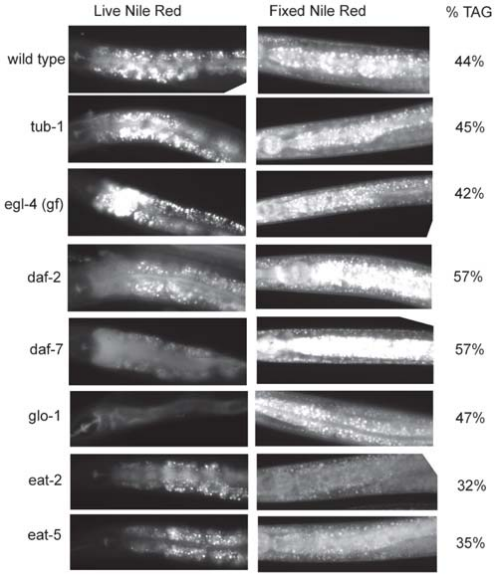
Comparison of live Nile Red staining, fixed Nile Red staining, and triacylglycerol stores in wild type and mutants. Triacylglycerol (TAG) stores were determined by TLC/GC of lipid extracts. %TAG refers to the percentage of total fatty acid detected in the TAG fraction. Anterior is to the left. Nile Red fed to live worms accumulates in gut granules, which are lysosome-related organelles. After worms are fixed in paraformaldehyde, the intensity and size of Nile Red staining-droplets correlates well with the biochemical determinations of TAG stores.

Because we found that wild-type worms grown on various bacterial strains do not show a difference in staining pattern visualized with Nile Red staining of live worms, but do have significant differences in fat stores as measured by TLC/GC lipid quantification or visualized with Nile Red in fixed worms, we examined a number of mutants that had previously reported fat storage differences measured by the levels of Nile Red brightness in live mutants. We found that Nile Red brightness in live worms did not always agree with fat storage levels measured by TLC/GC. In some strains Nile Red staining of live worms gives an overestimate (e.g. *egl-4(gf)* and *tub-1*), or an underestimate (*glo-1*, *daf-2*, and *daf-7*) of fat stores (Figure 2). When *C. elegans* are fed Nile Red, the dye accumulates in lysosome-related organelles called gut granules [14]. Staining procedures using fixed animals show a reproducible correlation between the brightness of Nile Red in fixed worms and fat storage levels measured by TLC/GC of lipid extracts (Figure 2). Because the fixed worms exhibit fat staining in the germline and hypodermis, as well as intestinal cells, and Nile Red staining in fixed worms is relatively uniform throughout the length of the worm, we believe that the fixation process allows the lipophylic dye access to fat stores throughout the animal. Our studies indicate that this technique will provide a more accurate visualization of fat stores in *C. elegans* than the widely-used technique of Nile Red staining of live worms.

### Lifespan is not markedly affected by bacterial diets

Many long-lived mutants of *C. elegans* exhibit metabolic changes that affect fat storage. For example, insulin receptor *daf-2* mutants have increased fat stores and a long lifespan [6], [15], while *eat-2* mutants and certain growth conditions causing caloric restriction, lead to increased lifespan but have decreased fat stores [16]. Because OP50, HB101, HT115, and DA837 strains had significant affects on fat storage, we examined mean and maximum lifespan of wild-type *C. elegans* growing on the four different bacterial strains. We found that even though fat storage levels differ depending on the strain of *E. coli* in the diet, the lifespan is not appreciably affected (Table 1).

**Table 1:** Lifespan analysis of wild-type *C. elegans* growing on four *E. coli* strains.

Bacterial Food	Mean lifespan (+/− SEM)	Max lifespan (+/− SEM)
OP50	12.7 (1.1)	26.6 (1.8)
HB101	14.3 (1.2)	26 (4.4)
HT115	14.5 (0.5)	24.5 (2.1)
DA837	12.8 (1.1)	23.3 (1.9)

### Carbohydrate levels in four dietary bacterial strains inversely correlate with fat stores in *C. elegans*, but do not appear to directly regulate fat storage

**Figure 3:**
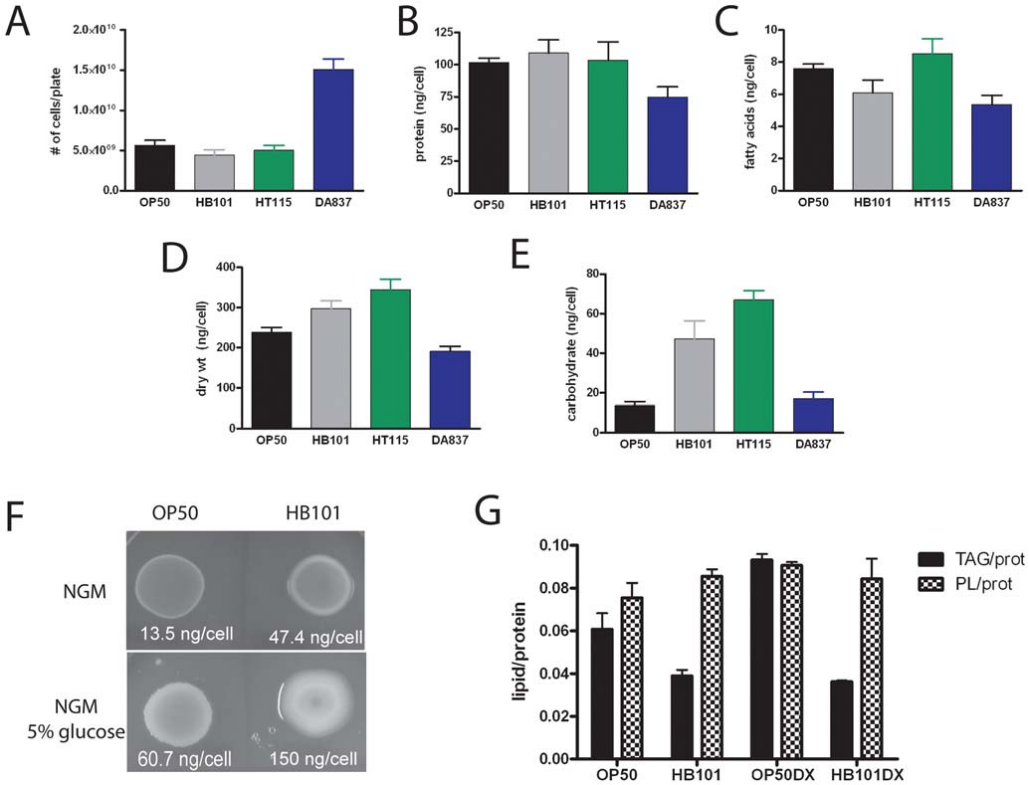
Characterization of cell number, dry weight, and macronutrient composition of *E. coli* lawns. Error bars are SEM, n = 3−5 independent growths. (A) Average number of viable bacterial cells in 3-day old lawns of *E. coli* washed off of 6 cm NGM plates. (B) Protein levels (normalized per cell) in four strains of *E. coli*. (C) Fatty acid levels (normalized per cell) in four strains of *E. coli*. (D) Average dry weight of bacterial lawns (normalized per cell) in four strains of *E. coli*. (E) The amounts of carbohydrate/cell vary significantly in the four *E. coli* strains. Total sugars in hydrolyzed *E. coli* lawns were determined by the Anthrone method. Error bars are SEM, n = 3−5 independent growths. (F) Addition of 5% glucose to plates leads to increased carbohydrate accumulation in bacteria and changes the morphology of bacterial lawns. (G) Growing bacteria on high glucose media increases carbohydrate levels in bacteria but does not cause decreased triacylglycerol stores in *C. elegans*. Error bars are standard deviation, n = 2−5 independent growths.

To investigate the basis of the fat storage differences in wild-type worms feeding on the four *E. coli* strains, we examined whether there were measurable differences in lawn density or macronutrients in the four *E. coli* strains that may contribute to differential fat stores in *C. elegans*. We first enumerated the number of cells on a bacterial lawn used in typical *C. elegans* experiments by counting colonies of serial dilutions of *E. coli* lawns washed off of standard 6 cm growth plates. In addition, we measured dry weight of the entire lawn, and determined protein, fatty acid, and total carbohydrate levels in the various strains. Even though HB101 forms visually thicker lawns than the other strains, we found that these lawns contain similar numbers of bacterial cells as OP50 and HT115 lawns (Figure 3A). In contrast, the DA837 lawns contained 2–3 times more cells, even though the appearance of the lawn is indistinguishable from OP50 lawns. Our results show that the major macronutrient in *E. coli* is protein, consistent with the reported composition of *E. coli* strain B grown in liquid culture [17]. In addition to having more cells per lawn, the DA837 strain showed lower protein, fatty acid, and dry weight per cell, indicating smaller cell size (Figure 3B–D). Among the remaining three strains, we measured similar levels of protein and fatty acids per cell (Figure 3B and 3C). The major difference in macronutrient composition among the four strains is that HB101 and HT115 contained 3–5 fold higher total carbohydrate levels than OP50 and DA837 (Figure 3E). The higher carbohydrate content may be due to the presence and extent of the extracellular capsule excreted by some strains of *E.coli*. This capsule is a protective structure made of colanic acid, a complex carbohydrate [18]. Thus, there appeared to be an inverse correlation between carbohydrate levels in the dietary *E. coli* strain and TAG stores in *C. elegans*.

To determine if dietary carbohydrates regulate fat storage in *C. elegans*, we altered the carbohydrate composition of the OP50 and HB101 bacteria strains by adding 5% glucose to the agar plates. Addition of glucose resulted in increased concentration of cellular carbohydrates in both bacterial strains, from 13.5 ng/cell to 60.7 ng/cell in OP50 and an increase from 47.4 ng/cell to 150 ng/cell in HB101, resulting in thicker lawns in both strains (Figure 3F). However, we found that the increased glucose content of the HB101 strain did not change TAG composition of nematodes, and increased glucose in OP50 resulted in slightly increased TAG storage in nematodes (Figure 3G). These experiments indicate that it is unlikely that carbohydrate content of dietary *E. coli* is regulating fat stores.

### Fatty Acid composition differences in *C. elegans* reflect the fatty acid composition in dietary bacteria, but do not correlate with fat storage

**Figure 4:**
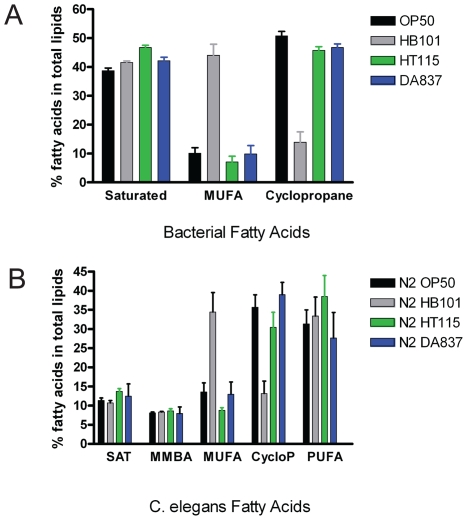
Fatty acid composition differences in *E. coli* strains and *C. elegans*. (A) The relative proportions of saturated, monounsaturated (MUFA), and cyclopropane fatty acids in four *E. coli* strains. *E. coli* lawns were washed off of NGM plates. Pelleted bacteria were derivatized to produce fatty acid methyl esters (FAMEs) for gas chromatography analysis. Error bars are standard deviation, n = 4−5 independent growths. (B) The relative proportion of saturated (SAT), monomethyl branched chain (MMBA), monounsaturated (MUFA), and polyunsaturated (PUFA) fatty acids in wild-type *C. elegans* raised on four *E. coli* strains. Error bars are standard deviation, n = 4−5 independent growths.

We then examined the fatty acid composition of the four bacterial strains and the fatty acid composition of the total lipids, as well as the TAG and phospholipid fractions in the worms. We found significant differences in the fatty acid composition of HB101 compared to the other three strains (Figure 4A). HB101 has higher levels of monounsaturated fatty acids (palmitoleic (16∶1) and vaccenic acids (18-1n-7), and reduced levels of cyclopropane fatty acids. The cyclopropane fatty acids are produced by bacteria during stationary culture and, theoretically, the differences among the strains may be accounted for by the activity of one gene, cyclopropane synthase, which converts monounsaturated fatty acids into cyclopropane fatty acids [19]. The worm lipids reflect their dietary lipids, with higher monounsaturated fatty acids levels accumulating in worms feeding on HB101 and higher cyclopropane levels accumulating in worms feeding on the other strains (Figure 4B). However, these fatty acid composition changes do not correlate with fat stores, because nematodes growing on HT115 show similar fatty acid composition to nematodes growing on OP50, yet their levels of fat storage differ significantly.

**Figure 5:**
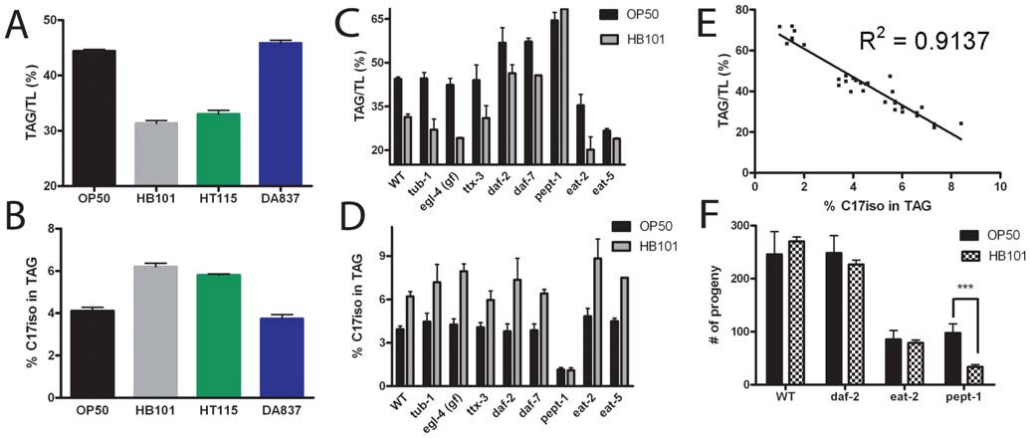
Relationship between branched chain fatty acid C17iso levels in triacylglycerol stores and total fat stores. (A) The percent of total fatty acids in triacylglycerol (TAG) fractions of wild-type young adults *C. elegans* feeding on four *E. coli* strains. Error bars are SEM, n = 3−5 independently grown samples. (B) The % of C17iso in triacylglycerol (TAG) fractions of wild-type young adults *C. elegans* feeding on four *E. coli* strains. Error bars are SEM, n = 3−5 independently grown samples. (C) The % of total fatty acids in TAG fractions measured in young adult wild type (WT) and various mutants. Although fat storage in many strains is greater or less than wild type, fat stores in most strains were reduced when grown on *E. coli* HB101 vs. OP50. Only *pept-1* mutants, defective in intestinal peptide transport, show no significant difference in fat stores when grown on OP50 and HB101. Error bars are standard deviation, n = 2−4 independently grown samples. (D) The % of C17iso in TAG fractions in various mutant *C. elegans* grown on OP50 and HB101. Error bars are standard deviation, n = 2−4 independently grown samples. (E) Inverse correlation between %C17iso in TAG and % of total fatty acids in TAG. Data points shown are 3–4 independent growths of wild-type worms on OP50, HB101, DA837, HT115 in addition to *pept-1* grown on OP50 and HB101. (F) Brood size is reduced in *pept-1* animals growing on HB101 compared to OP50, while brood size does not depend on dietary *E. coli* in wild type, *daf-2* or *eat-2* mutants. Error bars are standard deviation, n = 15 individuals of each genotype on each food source.

We then examined the fatty acid composition of phospholipid and TAG fractions to determine whether the relative fatty acid levels of any of the other worm fatty acids corresponded to TAG levels. We found that in the TAG fraction, the composition of one monomethyl branched-chain fatty acid, 15-methylpalmitic acid (C17iso), corresponds inversely to TAG levels in worms (Figure 5A and 5B). None of the other *C. elegans* fatty acids showed any type of correlation between TAG levels and fatty acid composition. It is important to note that C17iso is not a dietary nutrient, because the four *E. coli* strains that we examined to do not synthesize this fatty acid. This fatty acid is synthesized *de novo* by *C. elegans* [20], with the proposed pathway starting with a branched-chain alpha-keto acid derived from the amino acid leucine [21], [22].

### Differential fat storage still occurs in mutants defective in sensory pathways

To test if neuroendocrine pathways are necessary for the differential fat storage in worms feeding on different bacteria, we measured fat stores in the insulin receptor mutant *daf-2(e1370)* as well as the TGF-β mutant *daf-7(e1372)* strains. Fat stores in *daf-2* and *daf-7* mutants were high compared to wild type feeding on OP50 as well as on HB101, although in both mutant strains, fat stores in worms feeding on HB101 were lower than the fat stores in worms feeding on OP50, indicating that insulin and TGF-β sensory pathways are not necessary for differential fat storage on the two bacterial strains (Figure 5C). Food seeking behaviors that mediate dietary choice are regulated by the AIY neurons [10]. The *ttx-3* gene encodes a LIM homeodomain transcription factor required for the differentiation of AIY interneurons [23]. We found that, like wild type, *ttx-3* mutants stored less TAG when feeding on HB101 than on OP50, indicating that AIY interneurons, critical for mediating thermotaxis and food-seeking behaviors, are not required for mediating differential fat storage (Figure 5C).

We also examined two other mutants reported to contain high fat stores. One strain carries a mutation in *tub-1*, which is homologous to one of the few single-gene mutations that cause obesity in mice, *Tub* [24]. Another gene, *egl-4*, shows a bright Nile Red phenotype in gain-of-function mutants [25]. Even though our live staining experiments verified previous reports of bright Nile Red staining of live worms is increased in *tub-1* and *egl-4(gf)* mutants [26]–[29], we found that measurements of lipids consistently showed wild-type levels of TAG accumulation in both mutants feeding on OP50 and HB101. Two independently isolated *tub-1* mutant strains (*nr2004* and *nr2044*) both showed wild-type TAG accumulation. Also, like wild type, the levels of TAGs on OP50 were considerably higher than the levels on HB101 in both *egl-4(gf)* and *tub-1* mutants (Figure 5C).

Finally, we examined several eating-defective mutants and found that both *eat-2* and *eat-5* mutants stored lower fat on both bacteria (Figure 5C). Taken together, these studies show that sensory and feeding pathways necessary for the regulation of fat storage on OP50 are also necessary for wild-type levels of fat stored on HB101, yet these pathways are not necessary for distinguishing the difference between the two strains.

### Peptide transport in the intestine is necessary for fat storage differences

The only strain examined in this study that showed no difference in levels of fat storage when growing on OP50 and HB101 was a mutant *pept-1*, previously called *opt-2* and *pep-2*, which carries a deletion in a gene encoding an intestinal peptide transporter [30]. This gene was previously identified as a low-fat gene by live Nile Red staining [26]. Our measurements of TAG levels, however, revealed that this strain stores very high levels of fat, even when growing on HB101. Our lipid analysis also revealed fatty acid composition differences in *pept-1* mutants compared to wild type, with decreased amounts of monomethyl branched-chain fatty acids C15iso and C17iso as well as decreased levels of polyunsaturated fatty acids compared to wild type (Table 2). The fatty acid composition of *pept-1* mutants is similar to that reported by others [31], [32]. The levels of C17iso in TAGs, which are higher in most strains feeding on HB101, and therefore correlate inversely to overall TAG stores, were equally low in *pept-1* mutants feeding on OP50 and HB101 (Figure 5D). We found a significant inverse correlation of C17iso vs. %TAGs (R^2^ = 0.91) when examining the data set that included wild-type worms grown on the four bacterial strains and *pept-1* worms grown on OP50 and HB101 (Figure 5E).

**Table 2:** Fatty acid composition of total lipids of wild type and *pept-1*.

Fatty Acid	Wild type OP50	*pept-1* OP50	Wild type HB101	*pept-1* HB101
14∶0	1.1	1.5	0.8	1.1
C15iso	4.6	1.3	4.1	1.7
16∶0	4.3	6.8	3.9	6.7
C17iso	3.7	1.1	4.4	1.2
17Δ	18.9	26.7	9.5	22.0
18∶0	6.0	4.6	5.8	4.2
18∶1 Δ 9	3.3	2.6	3.4	4.2
18∶1 Δ 11	8.7	14.0	28.0	27.6
18∶2	5.5	3.2	6.8	5.1
19Δ	17.4	17.8	2.5	3.0
20∶3	3.8	3.3	3.5	2.5
20∶4n−6	1.3	1.1	1.7	1.6
20∶4n−3	4.5	2.6	3.6	1.9
20∶5	14.4	8.2	17.0	7.0

Data are weight percentages of total worm fatty acids measured by gas chromatography.

Abbreviations: C15iso, 13-methyltetradecanoic acid; C17iso, 15-methylhexadecanoic acid; 17Δ, cis-9,10-methylenehexadecanoic acid; 17Δ, cis-11,12-methyleneoctadecanoic acid.

### Reduced fertility in *pept-1* growing on HB101

Finally, we asked whether the differences in fat stores in worms grown on OP50 or HB101 affected reproductive success by counting the number of live progeny produced from individuals of various genotypes raised on either HB101 or OP50 *E. coli* lawns. We found that for wild type, as well as for *daf-2* and *eat-2* mutants, similar numbers of offspring were produced regardless of the food source. For *pept-1*, however, there was a significant reduction of progeny production in worms growing on HB101 compared to OP50 (Figure 5F). These results suggest that the range of TAG storage levels in wild type and *daf-2* on either food source are adequate to ensure efficient progeny production, but the feeding defects in *eat-2* and the peptide transport defects of *pept-1* may prevent adequate assimilation of nutrients and, consequently, reduced progeny production. Notably, in HB101, *pept-1* nematodes store greater than twice as much fat as wild type on HB101, but only produce 13% of wild-type brood size.

## Discussion

Obesity is a disorder in energy homeostasis that develops when energy intake exceeds energy expenditure. In order to prevent and treat obesity, it is important to develop a deeper understanding of the effects of dietary macronutrients on energy regulation pathways. We demonstrate that fat storage in *C. elegans* depends on the particular strain of dietary *E. coli* upon which it is feeding. In the wild, *C. elegans* is likely to feed on a wide range of bacterial species. Some bacterial species are pathogenic [33], [34], and worms can learn to avoid pathogenic food sources [13]. Given a choice, *C. elegans* chooses food certain bacteria over others [35]. Worms tend to leave undesirable bacteria food by engaging in increased roaming behavior, and this behavior depends on AIY interneurons [10]. Food choice studies have shown that compared to HB101, DA837 is considered to be a less desirable food, with cells that tend to clump together and may be difficult to ingest [10].

We suspected that differences in macronutrients of bacterial strains may be responsible for the range of fat stores observed in *C. elegans* feeding on various strains. We found differences in fatty acid composition, as well as differences in carbohydrate content among the four *E. coli* strains. Our analysis showed that fatty acid composition differences in dietary *E. coli* do not correlate with fat storage levels in *C. elegans*. Higher carbohydrate content of HB101 and HT115 correlates inversely with fat content, however, increasing carbohydrate content in HB101 and OP50 did not lead to a reduction in fat content, indicating that carbohydrate levels in bacteria per se do not dictate fat storage levels.

Analysis of TAG stores in a range of mutants indicated that sensory pathways are not necessary to store fat differentially on OP50 and HB101 food. Mutants defective in insulin signaling and TGF-β signaling both accumulate higher fat stores than wild type on both types of bacteria, however the mutants both accumulate less TAG when feeding on HB101 than on OP50. Furthermore, the *ttx-3* mutants, which are incapable of differentiating AIY interneurons critical for multiple sensory pathways, also accumulate less TAG when feeding on HB101 than on OP50. Only one mutant, *pept-1*, showed equally high fat stores when grown on both OP50 and HB101. This mutant is deficient in a peptide transporter expressed in the intestine [30]. Recent work demonstrates that even though endogenous fat synthesis is reduced in *pept-1* mutants, these worms accumulate high levels of fat due to accelerated uptake of dietary fatty acids [32]. This uptake is presumed to occur by way of a flip-flop mechanism that is dependent on intracellular and extracellular pH differences that are exacerbated in *pept-1* mutants.

Feeding behavior may also contribute to differential fat stores. A recent study showed that worms feeding on HB101, considered a high-quality food that is easy to ingest, entered into periods of quiescence, characterized by cessation of movement and pharyngeal pumping [36]. High-fat mutants such as *daf-2* and *daf-7* show reduced quiescence on HB101 [36]. Thus, wild-type nematodes growing on OP50, as well as *daf-2* and *daf-7* mutants growing on HB101, spend less time in quiescent states than wild-type worms growing on HB101. The reduced quiescence means more time is spent actively feeding, which correlates with higher fat stores. This suggests that the variation in fat stores in *C. elegans* growing on different *E. coli* strains may be due to the presence or lack of quiescence behavior. It is not known whether particular nutrients affect quiescence behavior.

Our TLC/GC analysis revealed that two mutant strains, *tub-2* and *egl-4(gf)*, which had been reported have high fat stores based on Nile Red staining of live worms [26]–[29], actually have wild type levels of fat stores. In addition, live Nile Red staining underestimates fat stores in *glo-1*, *daf-2*, and *daf-7* mutants. Furthermore, the *pept-1* mutant, which was identified as a low fat mutant in a Nile Red screen [26], actually has very high fat stores. These findings, together with the finding that live worms accumulate Nile Red dye in lysosomal compartments [14], cast doubt regarding utility of live assays using Nile Red for the determination of fat stores. However, after worms are fixed, Nile Red reveals lipid droplets in the intestine, hypodermis, and in the germline. The size of the droplets, as well as the intensity of staining, correlates well with TLC/GC analysis of fat stores in various mutants. A recent paper reported that a similar fixation procedure and staining with Oil-Red-O dye produced fat straining patterns that correlated with their quantitative biochemical lipid analysis [37]. Furthermore, the authors observed higher fat stores in wild-type worms grown on OP50 compared to HB101, consistent with results presented in this study.

An intriguing finding from this work is the inverse correlation between 15-methylpalmitic acid (C17iso) levels in TAGs and fat storage levels in wild-type worms raised on four different bacterial strains. C17iso is proposed to act as a chemical/nutritional indicator of the metabolic state of *C. elegans* [31]. C17iso is the final step of mmBCFA synthesis that initiates with a branched-chain alpha-keto acid precursor derived from the amino acid leucine [21]. Therefore, C17iso levels may reflect the levels of essential dietary amino acids. A recent study found that dietary leucine specifically rescues starvation-induced death in *gpb-2* mutants, and that dietary leucine suppresses starvation-induced stress and lifespan extension in wild-type worms [38], demonstrating the importance of this amino acid in regulating dietary responses in *C. elegans*. Future studies investigating the precise amounts of particular amino acid species in the dietary *E. coli* strains as well as in the worms feeding on them may provide further insight on the role of leucine in the regulation of fat storage.

## Materials and Methods

### *C. elegans* and *E. coli* strains and culture

*C. elegans* and *E. coli* strains used in this work were obtained from the Caenorhabditis Genetics Center. *E. coli* strain HT115 was from the Ahringer RNAi library purchased from Geneservice, Ltd, Cambridge, U.K. [39]. The following mutant strains and alleles were used: N2 (wild type), CB1372 *daf-7(e1372)*, CB1370 *daf-2(e1370)*, DA521 *egl-4(ad450)*, GH10 *glo-1(zu437)*, DA1402 *eat-5(ad1402)*, DA465 *eat-2(ad465)*, RB2742 *pept-1(lg1601)*, *tub-1(nr2004)*, *tub-1(nr2044)*. Worms were grown on NGM agar [9] at 20°.

*E. coli* strains used in this study:

**OP50.** A uracil auxotroph derived from *E. coli* B [9].

**HB101.** An *E coli* K12 x B hybrid, *mcr*B *mrr hsd*S *leu*B6 *sup*E44 *ara*14 *gal*K2 *lac*Y1 *pro*A2 *rps*L20(Smf) *xyl-5 mtl*-1 *rec*A14 [11].

**HT115 (DE3).** Derived from E. coli K12, F-, mcrA, mcrB, IN(rrnD-rrnE)1, rnc14::Tn10 (DE3 lysogen: lavUV5 promoter –T7 polymerase) [12], [40].

**DA837.** Derived from OP50 [41].

For bacterial cultures used to seed worm plates and for nutrient composition analysis, *E. coli* strains were grown overnight in LB agar at 37 degrees without shaking. NGM plates (6 cm) were seeded with 0.3 ml of overnight bacterial culture and plates were allowed to dry at room temperature for 2–3 days.

#### Lipid analysis of *C. elegans*

Early embryos were isolated from gravid adults by alkaline hypochlorite [9] and plated on nematode growth media (NGM). For each biological replicate, approximately 20,000 young adult nematodes were harvested, washed, and aliquots were removed for protein determination. The remaining nematodes were extracted overnight at 4° with chloroform:methanol (1∶1). The extract was washed with 0.2 M H3PO4, 1 M KCl and lipids were recovered in the chloroform phase and dried under argon. Neutral lipids were separated by thin layer chromatography on Silica gel plates as described in [42]. Triacylglycerol and phospholipid fractions were scraped for fatty acid methyl ester derivatization and analyzed by gas chromatography [43].

#### Nile Red staining of *C. elegans*

Live Nile Red staining of *C. elegans* was performed as described in [26]. Fixed Nile Red staining of *C. elegans* used a modified Sudan Black staining protocol [6]. Approximately 500–1000 nematodes are suspended in 1 ml of water. 50 µl of freshly prepared 10% paraformaldehyde solution is added, mixed, and worms are immediately frozen in briefly in liquid nitrogen. The worms are then subjected to two freeze/thaw cycles, taking care not to completely thaw the animals between cycles, after which worms are allowed to settle and the paraformaldehyde solution is removed. One ml of 1 µg/ml Nile Red in M9 is added to the worm pellet and incubated for 15–30 minutes at room temperature, with occasional gentle agitation. Worms are allowed to settle, washed once with M9 buffer, and allowed to settle again. After most of the staining solution is removed, the fixed worms are mounted onto 2% agarose pads for microscopic observation and photography. Nile Red images were acquired using identical settings and exposure times to allow direct comparisons.

#### Lifespan analysis of *C. elegans*

Lifespan analysis was carried out at 20°with worms maintained for several generations at 20° on consistent dietary bacterial strains. L4 worms were transferred to fresh plates at the beginning of the experiment, day 0 [15]. No FUdR or antibiotics were included in the plates. Worms were transferred to fresh plates daily until they stopped laying eggs, after which they were transferred every 4–5 days. Worms were scored daily for viability, and worms that crawled off the plate or burst at the vulva were excluded from the analysis. The mean and maximum lifespans were determined by the average of three to five independent trials, each using 40–100 animals.

### Macronutrient analysis of *E. coli*

For all assays, at least three independent bacterial growth experiments were performed, and each assay was repeated in triplicate for each growth.

Carbohydrate: Total sugars were determined with the anthrone method. Reducing and non-reducing sugars react with anthrone reagent under acidic conditions to yield a blue-green color [44]. Bacterial lawns were washed off of NGM plates with water. Aliquots of bacteria and glucose standards were added to 3 ml of anthrone solution (0.14% anthrone reagent in 60% sulfuric acid) and heated for 17 minutes at 90°C [45]. Absorbance of cooled samples was measured at 620 nm.

Protein: Protein was measured using bicinchoninic acid (BCA Protein Assay Kit, Thermo Scientific). Our protocol followed that described in [46], except that the assay was scaled accordingly to use a 10 µl aliquot of bacterial or nematode suspension.

Fatty Acids: Bacterial suspensions were pelleted and 15∶0 standard was added to the pellet. The mixture was subjected to simultaneous extraction and transmethylation by incubating for one hour at 70°C in 1 ml of 2.5% H_2_SO_4_ in methanol. Fatty acids were extracted with hexane and analyzed by gas chromatography as described in [43].

#### Fertility analysis of *C. elegans*

Total progeny were determined as described in [47].
